# Seed Transmission of *Soybean vein necrosis virus*: The First *Tospovirus *Implicated in Seed Transmission

**DOI:** 10.1371/journal.pone.0147342

**Published:** 2016-01-19

**Authors:** Carol Groves, Thomas German, Ranjit Dasgupta, Daren Mueller, Damon L. Smith

**Affiliations:** 1 Department of Plant Pathology, University of Wisconsin, 1630 Linden Drive, Madison, WI, 53706, United States of America; 2 Department of Entomology, University of Wisconsin, 1630 Linden Drive, Madison, WI, 53706, United States of America; 3 Department of Plant Pathology and Microbiology, Iowa State University, 351 Bessey Hall, Ames, IA, 50011, United States of America; Washington State University, UNITED STATES

## Abstract

*Soybean vein necrosis virus* (SVNV; genus *Tospovirus*; Family *Bunyaviridae*) is a negative-sense single-stranded RNA virus that has been detected across the United States and in Ontario, Canada. In 2013, a seed lot of a commercial soybean variety (*Glycine max*) with a high percentage of discolored, deformed and undersized seed was obtained. A random sample of this seed was planted in a growth room under standard conditions. Germination was greater than 90% and the resulting seedlings looked normal. Four composite samples of six plants each were tested by reverse transcription polymerase chain reaction (RT-PCR) using published primers complimentary to the S genomic segment of SVNV. Two composite leaflet samples retrieved from seedlings yielded amplicons with a size and sequence predictive of SVNV. Additional testing of twelve arbitrarily selected individual plants resulted in the identification of two SVNV positive plants. Experiments were repeated by growing seedlings from the same seed lot in an isolated room inside a thrips-proof cage to further eliminate any external source of infection. Also, increased care was taken to reduce any possible PCR contamination. Three positive plants out of forty-eight were found using these measures. Published and newly designed primers for the L and M RNAs of SVNV were also used to test the extracted RNA and strengthen the diagnosis of viral infection. In experiments, by three scientists, in two different labs all three genomic RNAs of SVNV were amplified in these plant materials. RNA-seq analysis was also conducted using RNA extracted from a composite seedling sample found to be SVNV-positive and a symptomatic sample collected from the field. This analysis revealed both sense and anti-sense reads from all three gene segments in both samples. We have shown that SVNV can be transmitted in seed to seedlings from an infected seed lot at a rate of 6%. To our knowledge this is the first report of seed-transmission of a *Tospovirus*.

## Introduction

In 2014, the U.S. harvested over 33 million ha of soybean with a total value of over $40 million dollars [1]. Soybean (*Glycine max*) is primarily grown for oil content (industrial and household) and meal production (animal feed). Its nitrogen-fixing capability makes it very important in cropping systems and crop rotations, particularly with corn (*Zea mays*). Many diseases cause production issues for soybean throughout the U.S., including several viruses. Viruses such as *Soybean mosaic virus* (SMV), *Bean pod mottle virus* (BPMV), and *Alfalfa mosaic virus* (AMV) commonly occur in soybean [2, 3].

Recently, a new soybean virus which causes vein necrosis was identified and named *Soybean vein necrosis virus* (SVNV) [4]. SVNV is a species within the genus *Tospovirus*. In the U.S. there have been very few instances where a *Tospovirus* was found to infect soybean. Nischwitz et al. [5] demonstrated that soybeans in Georgia were infected with *Tomato spotted wilt virus* (TSWV) and were asymptomatic. In other countries *Tospoviruses* have been more readily identified on soybean and include TSWV, *Tomato yellow ring virus*, *Groundnut ringspot virus*, *and Groundnut bud necrosis virus* [6]. SVNV is like other viruses in this genus in that its genome consists of a large negative-sense RNA component (L) and two smaller ambisense RNA components (M and S) that encode proteins in both the positive and negative-sense. The sizes of the M and S components are similar to other tospoviruses. The L component is the largest of the genus at 9,010 nucleotides [7]. The encoded proteins [nucleocapsid (N); nonstructural protein (NSs); glycoprotein (G_N_/G_C_); nonstructural protein (NSm); RNA-dependent RNA polymerase (RdRp)] are typical of the genus in general. However, SVNV does not conform to the typical sub-groups within the *Tospovirus* genus in that the homology of these proteins to other members of the genus is quite distinct. Tospovirus species within this genus are typically split between two distinct genetic clades called the ‘New World’ viruses and the ‘Old World’ viruses [7]. All viruses within the *Tospovirus* genus fall in these two sub-groups with the exception of SVNV and another closely related virus species, *Bean necrotic mosaic virus* (BeNMV) [7, 8]. SVNV was not identified as a pathogen of soybean until 2008 when it was documented in Tennessee, Arkansas, and several other southern states. More recently it has been found in north central U.S. states such as Wisconsin and Iowa [9]. Considering the uniqueness of SVNV and its relatively recent emergence as a soybean pathogen, it has been hypothesized that this virus has only recently adapted to soybean [7]. Several of the plant-infecting tospoviruses have very broad host-plant ranges, including SVNV. Zhou and Tzanetakis [4] identified several weed hosts capable of being alternative hosts for SVNV. Tospoviruses are transmitted exclusively by thrips, in a persistent propagative fashion (i.e., they replicate inside their thrips vector and are transstadially passed from molt-to-molt) [10]. SVNV can be transmitted by soybean thrips (*Neohydatothrips variabilis)* [4], but it is not known if other thrips species occurring on, or reproducing upon soybean can transmit the virus, or if there are other means of transmission.

Some soybean infecting-viruses like *Tobacco ringspot virus* (TRSV) can be transmitted via seed [11]. While the rate of seed transmission can be low, the impact on yield of this nepovirus can be high. Yield can be reduced as much as 100% through reduced pod set and seed formation. Seed from plants infected with TRSV often have higher total protein and lower total oil content than seed from non-infected plants [11]. It is generally accepted that members of the *Tospovirus* genus are not seed transmitted [12]. However, tospoviruses have been detected in pods of other legumes. Pappu et al. [13] demonstrated that TSWV, type species of the genus *Tospovirus*, localized to the peanut pod and testa using enzyme-linked immunosorbent assay (ELISA) and polymerase chain reaction (PCR) on plants that were symptomatic for TSWV infection. Sequences specific for TSWV were detected occasionally in cotyledons using PCR. Seedlings grown from seed harvested from both symptomatic and asymptomatic plants were tested for TSWV infection using ELISA, but no positively infected plants were identified [13]. These data suggest that in peanut, accumulation of the TSWV is localized to the shell and testa and is not passed to the progeny.

In 2013 a seed lot of commercial soybean seed from Nevada, Iowa that had a high level of discolored, deformed and undersized seed (‘damaged’ seed lot) was obtained. Virus infection was suspected, including SVNV. A separate seed lot from the same field that was normal in appearance (‘normal’ seed lot) was also acquired. In this paper, we provide evidence that i) the ‘damaged’ seed lot contained seed that was infected/infested with SVNV; ii) the SVNV-positive seed passed the virus to the emerged seedlings when planted. This is the first time that a Tospovirus has been found to be seed transmitted.

## Materials and Methods

### Soybean seed used in this study

**Fig 1:**
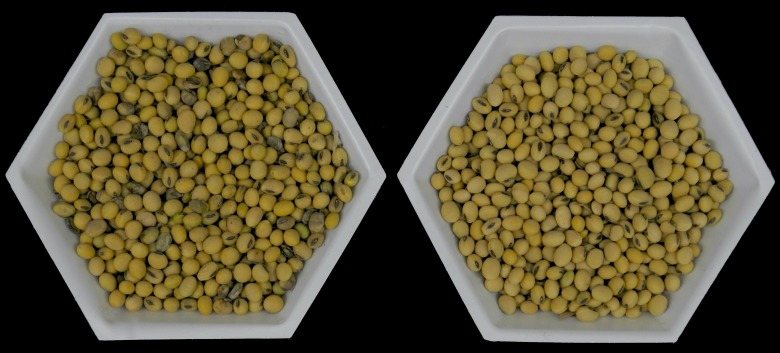
Visual appearance of ‘Damaged’ soybean seed lot and ‘normal’ soybean seed lot collected from the same field near Nevada Iowa in 2013. ‘Damaged’ seed is in the left tray and ‘normal’ seed is in the right tray.

The ‘damaged’ soybean seed (*Glycine max* (L.) Merr.) used in this study was hand harvested from a commercial soybean field (variety: Asgrow AG2433, Monsanto Company, St. Louis, MO) with symptoms indicative of virus infection in Nevada, Iowa in 2013. The geographical coordinates of the field where the sample was collected were 42.041262, -93.472741. The collection of seed was performed on private land with the consent of the farmer. In 2013, SVNV was widespread in Iowa and confirmed in soybean fields in every county by the Iowa State University Disease Diagnostic Clinic. The seed was maintained at room temperature in the University of Wisconsin-Madison Field Crops Pathology seed storage collection until use. An additional seed lot not visibly damaged and considered ‘normal’ in appearance, was also collected from the same field. This seed lot was used in the following experiments for comparison with the ‘damaged’ seed (Fig 1).

### Assessing Seed Quality Variables

Seed germination, 100-seed weight, protein content, oil content and fiber content were assessed for each of the two seed lots described above. Germination was determined by placing 10 seeds on filter paper saturated with de-ionized water and placed in a Petri plate. Each Petri plate was considered a technical replicate and five technical replicates were completed for each repetition. Two repetitions were conducted for each seed lot for a total of 100 seeds evaluated for each seed lot.

One hundred-seed weight (indicator of seed size) was determined by counting out five technical replicates of 100 seed for each seed lot. Two repetitions (2 separate days) were conducted for a total of 10 observations for each seed lot.

A grain analyzer (Foss 1241; Eden Prairie, MN) was used to determine total protein content, total oil content, and total fiber content of five technical replicates for each seed lot. The grain analyzer was programmed using a standard curve and five sub-samples were completed for each replicate.

All seed quality variables were subjected to a mixed model analysis of variance (ANOVA) with seed lot as the dependent variable and seed quality variables as the independent variables. Replicate and repetition were considered random effects. F-tests were performed at α = 0.05. All analyses were performed using SAS v. 9.4 (SAS Institute, Cary, NC).

Subsamples of seed from the ‘damaged’ and ‘normal’ lots were surface disinfested (1 min in 95% ethanol and 1 min in 10% Clorox) and plated onto potato dextrose agar (PDA) media amended with 25 μg/ml ampicillin, 10 μg/ml rifampicin, and 25 μg/ml streptomycin in Petri plates and incubated for 3–5 days at room temperature. Colonies of three different fungi were identified from seed sampled from the ‘damaged’ lot. Fungi were isolated and purified. Mycelia were subsequently collected from the culture plates and DNA extracted using the FastDNA spin kit (MP Biomedicals, Santa Ana, CA) according to the manufacturer’s instructions. DNA was subjected to PCR using ITS 4 and ITS 5 primers [14]. Resulting PCR products were purified for sequencing using the Wizard SV Gel and PCR Clean-Up System (Promega Corporation, Madison, WI). Sequencing was performed using ABI 3730xl DNA Analyzers (Applied Biosystems, Foster City, CA) at the University of Wisconsin-Madison Biotechnology Center. Sequences were subjected to BLASTn on GenBank for positive identification of fungal organisms.

### Initial virus detection assays

In April 2014, a random sample of soybean seed from the ‘damaged’ seed lot indicated above was planted in a growth room (14 hrs light/10 hrs dark, 22°C) at the University of Wisconsin-Madison. The growth room is located in the basement of Russell Laboratories and is lit by a combination of fluorescent and ultraviolet bulbs suitable for growing plants, and was isolated from any source of SVNV inoculum or thrips infestation. Soybean seed was planted into 32-cell propagation trays containing Sunshine Redi-earth Potting Mix (Sun Gro Horticulture, Agawam, MA). Germination of the seed was greater than 90% and the resulting seedlings appeared normal. Four composite samples of six plants each were tested using reverse transcription polymerase chain reaction (RT-PCR; described below) by selecting leaves (unifoliate or trifoliate) from each of six plants and combining them to create a composite sample. Subsequently, twelve individual plants (a single leaflet from each plant) were further tested after initial composite tests indicated the presence of SVNV.

### RNA extraction and RT-PCR

The harvested leaves were placed into individual plastic bags, flash-frozen in liquid nitrogen and stored at -80°C prior to RNA extraction. Approximately 200 mg of leaf or cotyledon tissue were ground in liquid nitrogen and RNA was extracted using the TRIzol® Plus RNA Purification Kit (Life Technologies, Carlsbad, CA) according to the manufacturer’s instructions. RNA quantity was estimated on a NanoDrop UV-Vis Spectrophotometer (Thermo Fisher Scientific, Inc., Waltham, MA). Approximately 1 μg of total RNA was used to generate first-strand cDNA with random or specific primers using the iScript™ Reverse Transcription Supermix for RT-PCR (Bio-Rad Laboratories, Hercules, CA) or Superscript III one step RT-PCR kit with platinum Taq DNA polymerase (Invitrogen/ Life Technologies, Carlsbad, CA) according to the manufacturer’s instructions. cDNAs were diluted ten- to twenty five-fold in nuclease free water for use in subsequent PCR.

Specific primers were used to amplify portions of all three genomic segments of SVNV (Table 1). Primers designed to amplify the gene encoding the entire nucleocapsid protein [6] were used in nested PCR reactions using SVNaV-f1/SVNaV-r1 and SVNaV-f2/SVNaV-r2 primer sets. Published primers were used to amplify regions of the SVNV L segment [7]. To amplify a portion of the M segment, primers were designed using PrimerQuest Software Package (IDT DNA Technologies, Coralville IA; Table 1). PCR were performed using GoTaq Green Master Mix (Promega Corporation, Madison, WI) containing: GoTaq DNA Polymerase in 1X Green GoTaq Reaction Buffer (pH 8.5), 200 uM dNTPS, and 1.5 mM MgCl_2_; 1 uM of each primer; and 5–7.5 ul cDNA in the total volume of 25 ul per reaction. PCR was performed under the following conditions in an Eppendorf MasterCycler Pro S programmable thermal cycler (Eppendorf AG, Hamburg, Germany): denaturing at 95°C for 2 min followed by 32–40 cycles of 30 sec denaturation at 94°C, 30 sec annealing at 52°C, and 1 min elongation at 72°C, followed by a final extension step at 72°C for five to ten min. Nested PCR (S segment) was performed as described above using specific internal primers (Table 1) and 5 μl of the first round PCR product as the template. Reaction conditions for all PCR was similar to that described above. Proper negative (water blanks) and positive (cDNA from a SVNV positive field isolate) controls were used in all PCR experiments. PCR fragments were visualized by electrophoresis in 1X TAE on a 1.2% agarose gel containing SYBR Safe DNA gel stain (Life Technologies, Carlsbad, CA), using PCR Marker (Promega Corporation, Madison, WI) or 1 kb DNA Ladder (Promega Corporation, Madison, WI) to estimate the size of the fragments. Select PCR fragments were purified for sequencing using the Wizard SV Gel and PCR Clean-Up System. Sequencing was performed using ABI 3730xl DNA Analyzers at the University of Wisconsin-Madison Biotechnology Center. Template RNA was also checked for the presence of endogenous DNA by using the above PCR primers and conditions. RNA from the initial extraction was used as template instead of cDNA as described above. Genomic DNA extracted from the same plant material was used as template and subjected to PCR using the primers described above and soybean genomic primers to rule out the presence of sequences homologous to SVNV that could be transcribed and result in false positives in the PCR experiments.

**Table 1:** Primers used to detect SVNV in soybean seedlings and product sequence identities as compared to known sequences in GenBank.

Primer Name	Sequence 5’-3’	Product size (approximate)	Sequence Identities of PCR Products^1^
**L segment**			
LdetF^2^	GAGCCCATAAACCTGTCTGC	297 nt	96–99%
LdetR^2^	TGCCATGATGTGCTCAGATT		
**M segment**			
MF-1681	GATGGTTTCTGGGTCAGAGATT	494 nt	97–99%
MR-2175	CACTTGGTCTTGTGCTGTTATTG		
**S segment**			
SVNaV-f1^3^	AGATATAAAGTTGAGACACTATC	939 nt	
SVNaV-r1^3^	TGCAACACATCCGGAACTCTG		
SVNaV-f2^3^	CCTGAATTCATGCCACAAACAGCAGG	845 nt	98–99%
SVNaV-r2^3^	TTAGCGGCCGCTAAACAGAAAACTCC		

^1^Sequences obtained from seed borne virus compared with SVNV sequences in GenBank.

^2^Zhou et al., 2011.

^3^Khatabi et al., 2012.

*F/f* forward primer (sense), *R/r* reverse primer (antisense), *nt* nucleotides

### Controlled environment studies

A thrips-proof cage large enough to contain three-32 cell propagation trays was used to cultivate and maintain plants in a controlled environment. The thrips proof cage was housed in an environmentally controlled growth room in the basement of Russell Laboratories, and was maintained at 22°C, under a 14 hr day/10 hr night photoperiod and was lit by a combination of fluorescent and ultraviolet bulbs suitable for growing plants. Seeds from the Nevada, Iowa seed lot were randomly planted in the trays, with no bias as to the physical appearance of the seed. The seed was planted into Sunshine Redi-earth Potting Mix, as described above. Once the plants emerged, and the first trifoliate began to open (usually 10–11 days post planting) one cotyledon from each sample plant was removed. Eight randomly selected plants (random number generator was used) from each tray were sampled. For each experimental run: a single tray (32 cells) represented a replication, thus, there were 3 replications per experimental repetition. Sample size was 8 plants per tray (sub-sample from the population) and 3 replications per experimental repetition, for a total of 24 plants per experimental repetition. Two repetitions were conducted. RNA was extracted from each plant and cDNA was synthesized and subjected to RT-PCR, as described above, in order to determine the presence of SVNV in all plants sampled for each repetition.

### Total RNA sequencing (RNA-seq)

Total RNA was extracted and quantified as described previously. The total RNA extracts from a composite sample (multiple soybean leaflets), and also from a SVNV-symptomatic leaflet collected from a Wisconsin field, both of which were PCR-positive using SVNV primers were sent to ProteinCT Biotechnologies LLC (Madison, WI) where they were treated with DNase and the total RNA was used to construct a micro RNA library using the SeqMatic Micro RNA Sample Preparation kit (SeqMatic LLC, Fremont, CA). The total RNA library was subjected to high throughput sequencing (1x50bp) using the Illumina platform (Illumina, Inc., San Diego, CA). RNA reads were quality-filtered to remove low quality and very short reads and to remove adaptors. The clean reads were then aligned to the published soybean genome (the v2.0 assembly, *Glycine max Wm82*.*a2*.*v1* http://phytozome.jgi.doe.gov/pz/portal.html#!info?alias=Org_Gmax) and to the SVNV genome (GenBank accession numbers, HQ728385.1, HQ728386.1, and HQ728387.1) using Bowtie short read aligner [15].

## Results and Discussion

### Seed Quality Assessment

Germination, 100-seed weight, total protein content and total fiber content were not significantly different between ‘normal’ and ‘damaged’ seed lots. Total oil content was significantly lower in the ‘damaged’ seed lot compared to the ‘normal’ seed lot (Table 2). These results suggest that virus infection, including infection by SVNV of parent plants may influence the chemical composition of soybeans. Decreased oil content is of concern as soybean is an oilseed crop, grown in part for industrial oil needs. The reduction in oil content and increase in protein content is consistent with previous work on soybean. Demski et al. [16] and Demski and Jellum [17] found that *Tobacco ringspot virus* alone, or in mixed infection with other viruses, typically reduced oil content of soybeans and increased total protein content. Furthermore, virus infection was found to change the fatty acid composition of soybean oil [17]. While we did not test the fatty acid content of the damaged seed examined here, trends in reduced oil content and increased protein in soybean seed infected with a systemic virus are similar to previous reports.

**Table 2:** Results of mixed model analysis of variance of seed quality variables of AG2433 soybean seed collected from visually ‘damaged’ and ‘normal’ locations in the same soybean field in Nevada Iowa, 2013.

	Germination (%)^a^	100-seed weight (g)^b^	Protein content (%)^c^	Oil content (%)^c^	Fiber content (%)^c^
‘Damaged’ seed	94.0	15.1	36.2	18.7	4.6
‘Normal’ seed	100.0	15.4	35.8	19.5	4.6
F-value^d^	0.08 ns	0.3 ns	2.7 ns	27.4 *	1.0 ns

^a^Germination was determined by recording germination of 10 seeds for 5 technical replicates for each seed lot on two different days (2 repetitions) for a total of 10 observations for each seed lot

^b^100-seed weight was determined by taking weights on 5 technical replicates for each seed lot on two different days (2 repetitions) for a total of 10 observations for each seed lot

^c^Protein, oil, and fiber content was determined by using a Foss 1241 grain analyzer. Grain samples of each seed lot were split into three technical replicates. The grain analyzer performed five sub-sample readings of each technical replicate.

^d^ns = not significant at *P* ≤ 0.05

* = significant at *P* ≤ 0.05

Fungi isolated from the ‘damaged’ seed lot included *Diaporthe phaseolorum*, *Alternaria alternata*, and *Fusarium equiseti* all with 98–99% homology to sequences in GenBank (*data not shown*). No pathogenic fungal organisms were isolated from the ‘normal’ seed lot. All three organisms isolated from the ‘damaged’ seed lot have been reported as pathogens of soybean [18]. *Diaporthe phaseolorum*, and *Alternaria alternata* have been implicated in causing pod blights and *Fusarium equiseti* may also be found on soybean plant parts including pods. These findings suggest that there might be correlation between virus infection and increased presence of fungal pathogens of seed, which further reduce overall seed quality. However, it is difficult to determine if virus infection increases susceptibility of seed to fungal infection or vice versa.

### SVNV positives in initial RT-PCR

**Fig 2:**
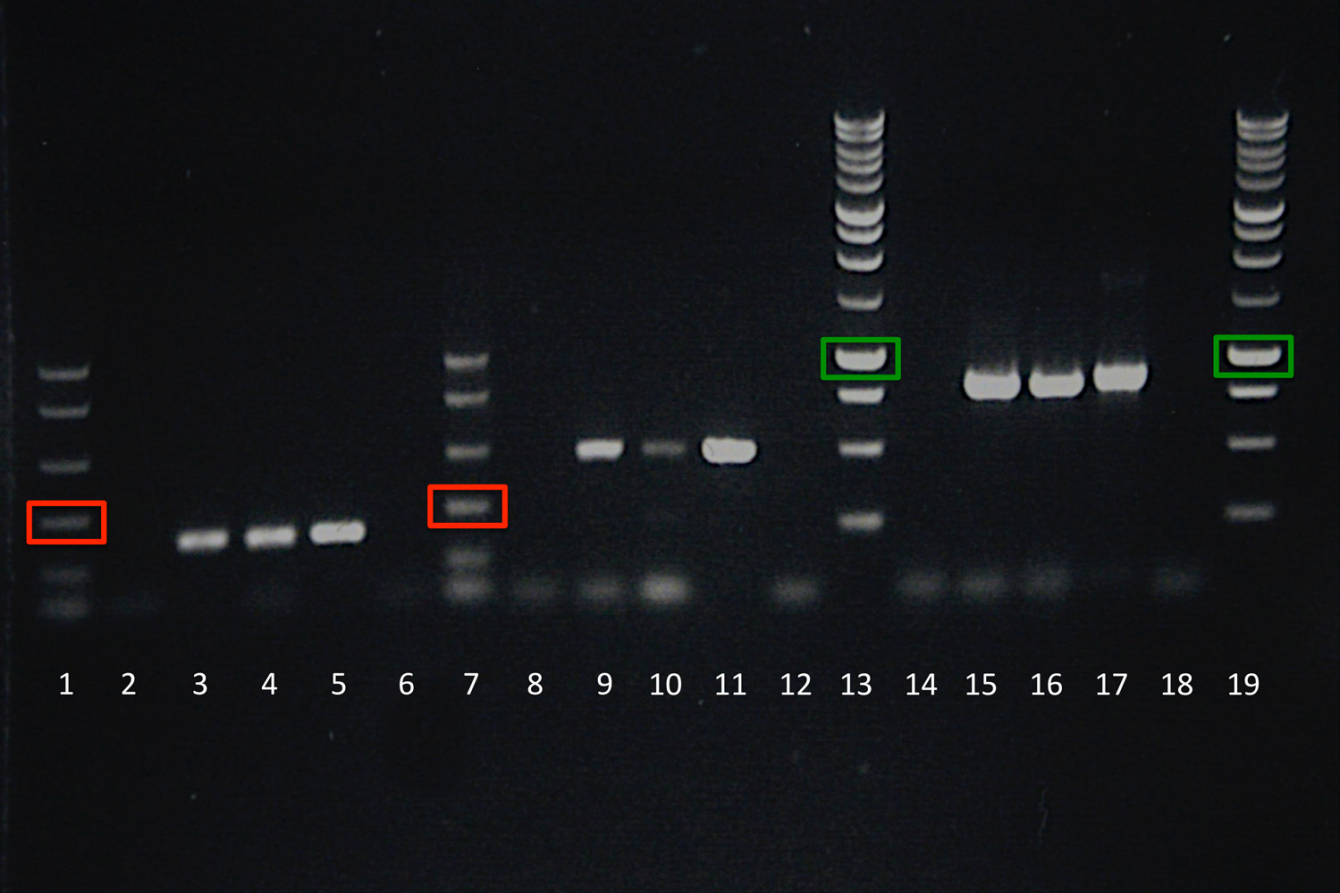
Electrophoresis results after RT-PCR using primers specific for L, M and S gene segments of *Soybean vein necrosis virus*. Lanes 1 and 7 –PCR Marker (red boxes indicate 300 bp fragment size); Lanes 13 and 19–1 kb ladder (green boxes indicate 1,000 bp fragment size); Lanes 2 and 6 –Negative (water) control with LdetF/LdetR primers; Lanes 8 and 12 –Negative (water) control with MF1681/MR2175 primers; Lanes 14 and 18 –Negative (water) control with SVNaV-F2/SVNaV-R2 primers; Lanes 3, 9, and 15 –Composite RNA extraction from multiple leaves of plants derived from the damaged seed lot and RT-PCR with LdetF/LdetR primers, MF1681/MR2175 primers, and SVNaV-F2/SVNaV-R2 primers, respectively; Lanes 4, 10, and 16 –RNA extraction from a single plant derived from the damaged seed lot and RT-PCR with LdetF/LdetR primers, MF1681/MR2175 primers, and SVNaV-F2/SVNaV-R2 primers, respectively; Lanes 5, 11, and 17—RNA extraction from a SVNV-symptomatic soybean plant from a field in Wisconsin in 2013 and RT-PCR with LdetF/LdetR primers, MF1681/MR2175 primers, and SVNaV-F2/SVNaV-R2 primers, respectively.

In April 2014, a random sample of soybean seed from the Nevada, Iowa ‘damaged’ seed lot was planted in the growth room to determine whether or not the resulting plants would be symptomatic for viral infection. Germination of the seed was greater than 90% and the plants looked normal. Leaves were selected from the flats of plants to test with RT-PCR in ‘composite’ sample (six plants were represented in each composite). RT-PCR products were obtained from two of the four composite samples tested in initial experiments using the nested primers for the SVNV S segment (Table 1). Fig 2 illustrates a composite sample extraction and RT-PCR amplification using the three primer sets (Lanes 3, 9, and 15). In subsequent experiments with individual plants from this same group of plants, two of 12 individual plants tested, yielded RT-PCR products with the nested primers (SVNaV-f1/SVNaV-r1 followed by PCR using SVNaV-f2/SVNaV-r2 primers). Fig 2 illustrates one of the single plant extractions and RT-PCR amplifications with the three primer sets, which is also compared to PCR amplification from RNA extracted from a symptomatic field isolate from Wisconsin in 2013 (Lanes 5, 11, and 17). The PCR products from the composite samples and the individual samples were sequenced and the resulting sequences were at least 98% identical to published SVNV S segment sequences including HQ728387 (Table 1). These results demonstrate that SVNV was present in leaves of the plants tested, and most likely moved from infested seed to emerging seedlings.

Further testing of plant material was also conducted using the enzyme-linked immunosorbent assay (ELISA) described by Khatabi et al. [6]. No positive confirmations of SVNV seed-transmission were identified using this approach, including confirmation from plant material previously found to be positive using RT-PCR. This could be due to extremely low virus titer in plants containing the seed-transmitted isolate of SVNV identified here. In previous work we found that RT-PCR using the N gene nested primers in Table 1 was a highly sensitive method for detecting SVNV in low copy number [19]. PCR protocols were consistently more reliable than ELISA for detection purposes for SVNV and were used for all subsequent experiments. PCR reactions using RNA as template instead of cDNA resulted in no products after gel electrophoresis for all but three samples. In those three samples, the bands (~150bp in size) did not correspond to the expected sizes for each primer pair. These small bands were excised and sequenced and checked using BLASTn. No matches were identified (data not shown). Therefore, no DNA contamination was identified in the RNA preparations used in these studies. Additionally, PCR reactions using DNA extracted from the same plant material resulted in no products after gel electrophoresis, indicating that transcribed viral DNA was not integrated in the plant genome.

### SVNV detection in controlled environment studies

After the initial detection of SVNV in plants grown in the growth room, additional measures were taken to further control the growing environment by restricting access to any possible thrips infestation. Subsequent controlled environment studies were performed in a thrips-proof cage to eliminate any possibility of SVNV infection due to thrips feeding. Yellow sticky traps used for catching insects were placed both inside and around the thrips-proof cage to monitor for any insect activity. Thrips cards were periodically examined for presence of thrips. Thrips were never trapped inside the thrips-proof cage. Occasionally eastern flower thrips (*Frankliniella tritici*) were trapped outside the thrips cage. Soybean thrips have been identified as vectors of SVNV [4]. It has not been demonstrated that eastern flower thrips can transmit SVNV.

A total of 48 plants were tested (8 plants x 3 replicates x two repetitions). Three plants from the 48 sampled were determined by RT-PCR (using primers as indicated in Table 1) to be infected with SVNV. These results confirm the presence of SVNV in the AG2433 seed lot examined. The mean proportion of plants infected with SVNV was therefore 0.06.

### RNA-seq results

**Fig 3:**
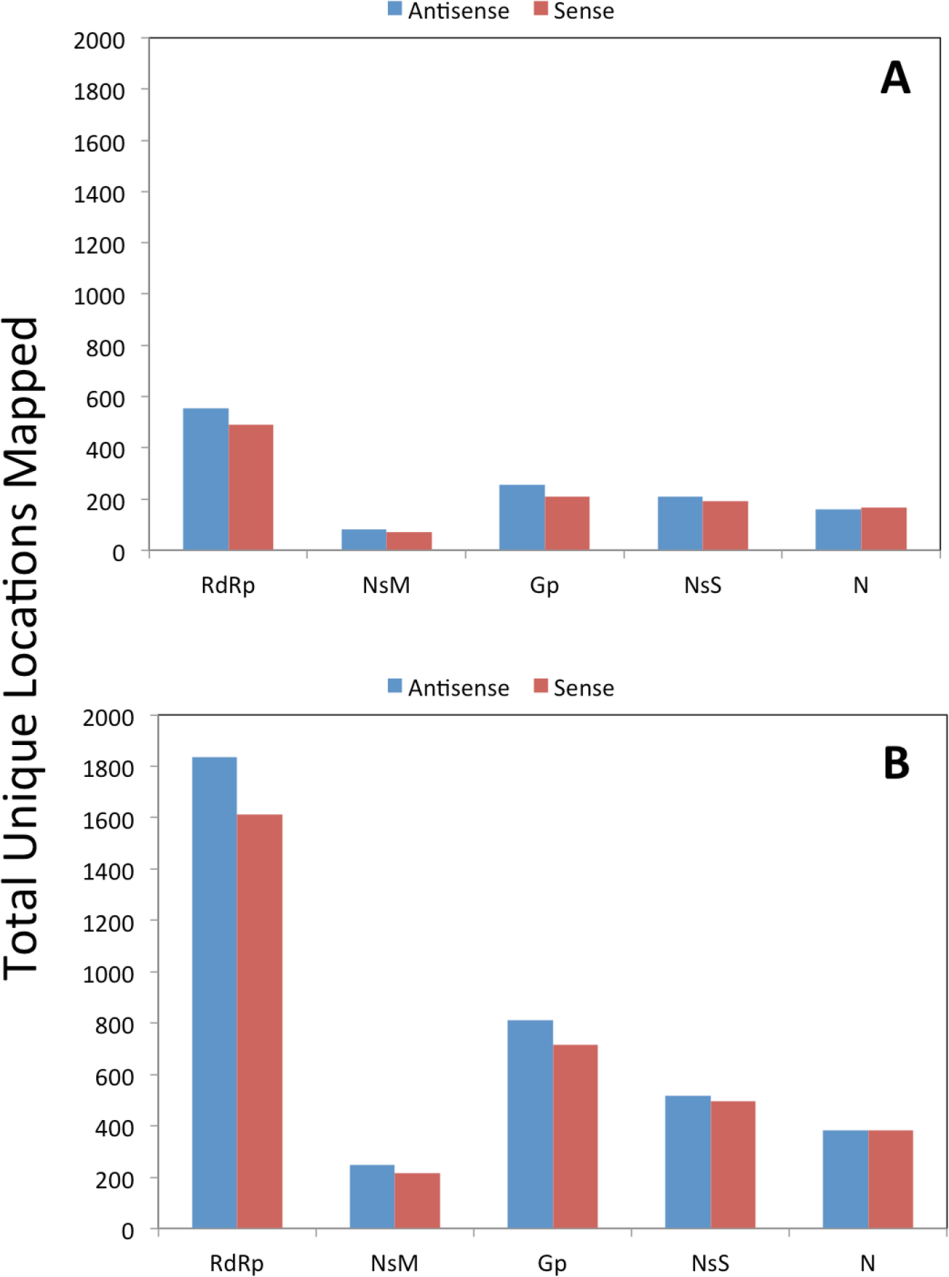
Number of sense and antisense reads for specific *Soybean vein necrosis virus* genes from a small RNA-seq analysis for A, using RNA extracted from a composite sample of multiple leaves from plants derived from the damaged seed lot and grown in a thrips-proof cage in a controlled environment or B, using RNA extracted from a symptomatic soybean sample collected from a field located in Lancaster, Wisconsin. RdRp = RNA-dependent RNA polymerase; Nsm = nonstructural protein, M; GnGc = glycoprotein precursor; NSs = nonstructural protein, S; N = nucleocapsid protein.

**Fig 4:**
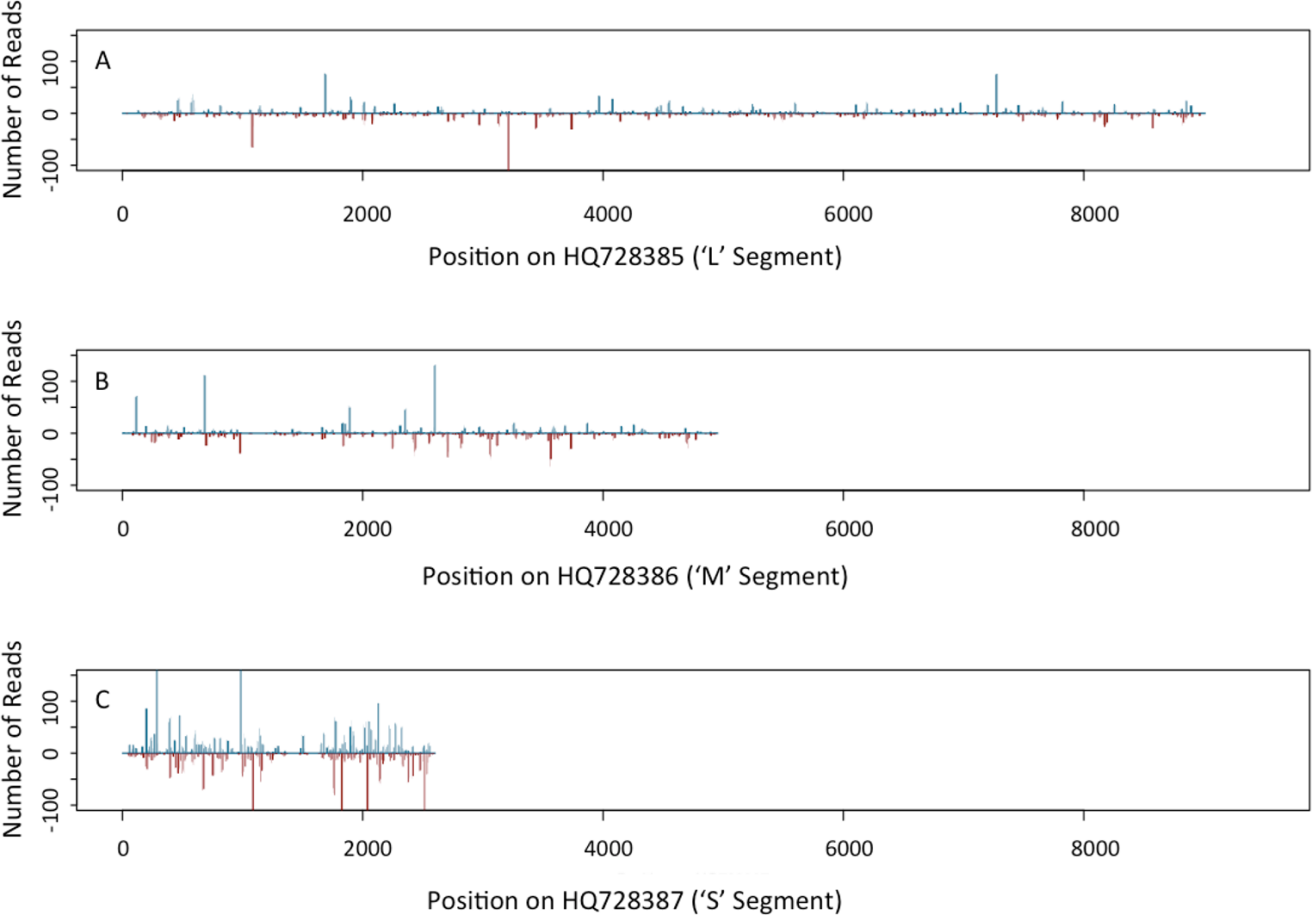
Coverage map from a small RNA-seq analysis showing number of reads on the L segment, M segment, and S segment of the *Soybean vein necrosis virus* genome for the composite sample. Analysis was conducted using RNA extracted from a composite sample of multiple leaves from plants derived from the damaged seed lot and grown in a thrips-proof cage in a controlled environment. Blue bars indicate sense reads, while red bars indicate antisense reads along the SVNV genome.

**Fig 5:**
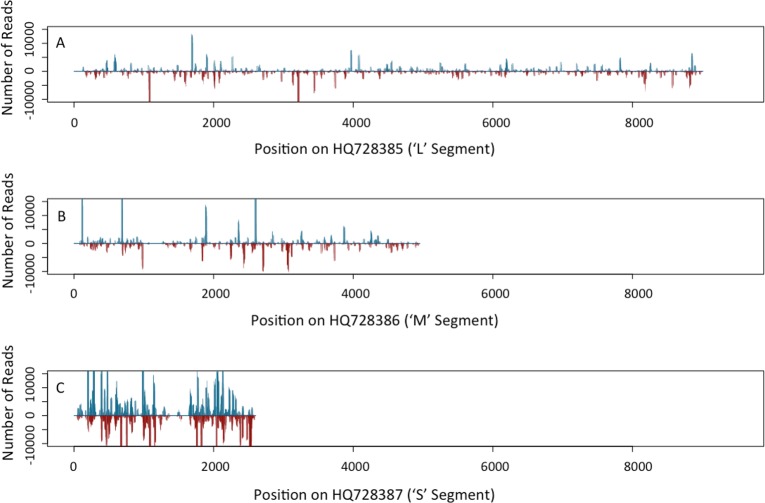
Coverage map from a small RNA-seq analysis showing number of reads on the L segment, M segment, and S segment of the *Soybean vein necrosis virus* genome for the symptomatic field sample. Analysis was conducted using RNA extracted from a symptomatic leaf sample collected from Lancaster, Wisconsin. Blue bars indicate sense reads, while red bars indicate antisense reads along the SVNV genome.

From the RNA-seq analysis, 1,790,826 total reads were obtained from the composite sample of plants from seed grow-outs, of which 9,376 reads (0.60%) mapped to the SVNV genome. For the symptomatic field sample, 6,457,522 total reads were obtained with 2,025,891 reads (35.10%) mapped to the SVNV genome. The total RNA extract from the composite sample that was PCR positive using SVNV primers, and the symptomatic field sample, were identified to have numerous reads of small RNA segments with sizes ranging from 18–23 nt, which would be appropriate for RNA viruses, and with hotspots in the 21–22 nt range. Further analyses revealed that both sense and antisense reads for specific genes for SVNV were identified for both the composite sample and the symptomatic field sample (Fig 3) and were spread throughout the entire three genomic segments of SVNV (Figs 4 and 5). Our intention in conducting the RNA sequencing experiment was not to do a thorough bioinformatics analysis of the response of soybean to SVNV infection but to use the data to establish the presence of the virus in the plant material sample subjected to analysis, as has been previously done [20, 21, 22]. The RNA used for library construction and sequencing for the seed-transmitted strain was from a composite sample so the number of infected leaves was likely a low number obviating the large number of viral reads needed for a more in depth analysis. However, the fact that the size distribution of the small RNAs mapping to the SVNV genome is consistent with that expected for siRNAs [23], and that the location of reads and pattern is similar to that of the symptomatic field isolate, indicates that the SVNV virus is present in the RNA extract of the composite sample taken from seed grown in isolation. In this study the size distribution of small RNAs clustered between 21 nt and 22 nt similar to that from TSWV-infected *Nicotiana benthamiana* and tomato [24]. The distribution of matches along the genomic and complimentary strands of all three viral RNAs, and their mapping to all viral open reading frames provides independent, corroborating evidence for the RT-PCR data indicating the presence of SVNV in plant material grown from seed. Furthermore, these results are consistent with the occurrence of virus replication in the emerging seedlings and systemic transport throughout the plant. Zhou and Tzanetakis [4] reported that soybean was a local-lesion host for SVNV and that the virus did not systemically infect this host. However, the isolate of SVNV present in the seed lot examined here, appears to be systemic without causing typical symptoms on soybean. Follow-up testing of newly developing leaves of plants previously found to be positive for SVNV seed-transmission have also yielded positive PCR products. In addition, roots from plants grown in isolation have been tested for SVNV and positive PCR products have been identified using the S genomic segment primers. Sequencing of the PCR product identified a 98% match to the known SVNV segment in GenBank. These results further substantiate the fact that the SVNV isolate found in the seed lot tested here is systemically transported in soybean plants. In a recent paper by Hajimorad et al. [25], they identified soybean plants with SVNV grown in a greenhouse. The SVNV-positive plants were grown from seed collected from the field. The authors postulate that the presence of SVNV in greenhouse grown plants was likely a result of feeding by overwintering, viruliferous adult thrips. However, three soybean varieties were grown in the same greenhouse and only two of the varieties were infected with SVNV. If viruliferous thrips were the source of SVNV inoculum, it would be presumed that all three varieties would have been infected, as genetic resistance toward SVNV is not known. In addition, distribution of viral symptoms should have been fairly uniform in this environment, if thrips were the source of inoculum. Based on the results of our study, we hypothesize an alternative explanation that the SVNV-infected greenhouse plants described by Hajimorad et.al. [25] likely could have arisen from seed-borne SVNV transmission.

### Conclusions

To our knowledge this is the first report of seed-transmission of a *Tospovirus*. We have shown that SVNV can be transmitted in seed and systemically transmitted to the emerging seedlings at a rate of approximately 6%. Previously, it was reported that this virus was not systemically transmitted in soybean. The seed-transmitted isolate of SVNV identified in this work causes no foliar symptoms on soybean. However, the seed lot containing the seed-transmitted isolate of SVNV was found to have reduced total oil and slightly elevated protein content. In addition, three fungal organisms were also found parasitizing the same seed. It is not clear if SVNV or mixed virus infections predispose seed to increased infection by fungal pathogens. These findings are especially important for soybean seed farmers who seek high quality, pathogen-free seed and who desire high oil output from a soybean crop. SVNV might also interact with other viruses in the same soybean plant to cause synergistic reactions that could result in increased damage to soybean. Therefore, identifying SVNV seed transmission and removing contaminated seed from a seed lot could help reduce damage caused by plant pathogenic virus synergism. Finally, it is possible that the asymptomatic, seed-transmitted isolate identified here could recombine with symptomatic isolates present in the field resulting in a more aggressive and destructive seed-transmissible strain that could cause significant damage to soybeans in the field. Therefore, it is important to identify seed lots with SVNV and remove contaminated seed before it makes it to breeding nurseries or farmer fields. Experiments are in progress to determine the additional agronomic importance and to determine the mechanism by which seed transmission of SVNV is occurring.
